# Total hip arthroplasty in a patient with congenital insensitivity to pain: a case report

**DOI:** 10.1186/1752-1947-6-190

**Published:** 2012-07-09

**Authors:** Mehmet Erdil, Kerem Bilsel, Yunus Imren, Hasan Huseyin Ceylan, Ibrahim Tuncay

**Affiliations:** 1 Orthopedics and Traumatology Department, Faculty of Medicine, Bezmialem Vakif University, Istanbul, Turkey

## Abstract

**Introduction:**

Congenital insensitivity to pain, a rare neurological entity, is characterized by varying degrees of sensory loss and autonomic dysfunction. Orthopedic manifestations of congenital insensitivity to pain include delayed diagnosis of fractures, nonunions, malunions, Charcot arthropathy, acro-osteolysis, avascular necrosis, osteomyelitis, heterotopic ossification and joint dislocations. We here report the case of a patient with congenital insensitivity to pain who had multiple lower extremity fractures at varying intervals, the most recent being a femoral neck fracture managed by total hip replacement. To the best of our knowledge, this is the first report of cementless hip arthroplasty in such a patient.

**Case presentation:**

A 37-year-old Caucasian woman was admitted to our hospital complaining of painless swellings in her lower limb and limping. She had been diagnosed with multiple lower extremity fractures at different times. On physical examination, we found multiple perioral mucosal ulcers, shortening of her nails and acro-osteolysis, a prematurely aged facial appearance, undersized skeletal structure, Charcot arthropathy of her right ankle, anosmia, insensitivity to temperature differences and evidence of mild intellectual disability. A right subtrochanteric femur fracture was treated with an intramedullary nail. Eighteen months later, she presented with similar symptoms and we diagnosed a right femoral neck fracture. We removed the nail and performed cementless total right hip arthroplasty.

**Conclusions:**

Congenital insensitivity to pain is a rare condition that is associated with severe orthopedic problems. This case report, which will be of particular interest to orthopedic surgeons, presents several difficulties in the management of patients with congenital insensitivity to pain and notes the importance of close follow-up and early recognition of complications. Cementless total hip arthroplasty may be a good therapeutic option for femoral neck fracture in these patients.

## Introduction

Congenital insensitivity to pain (CIP), a rare neurological entity, is characterized by varying degrees of sensory loss and autonomic dysfunction. This condition was first described by Dearborn in 1932 [1]. Dyck named this condition hereditary sensory autonomic neuropathy (HSAN) and categorized it into five different types [2]*.* Orthopedic manifestations of CIP include delayed diagnosis of fractures, nonunions, malunions, avascular necrosis, osteomyelitis, heterotopic ossification and joint dislocations [2-5]*.*

We here report a case of a 37-year-old Caucasian woman who had multiple lower extremity fractures at varying intervals. The aim of this case report is to illustrate the complex orthopedic problems of a patient who is insensitive to pain and has undergone multiple operations, the most recent being total hip replacement for a femoral neck fracture. To the best of our knowledge, this is the first report of hip arthroplasty in such a patient.

## Case presentation

Three years prior to her presentation with a fractured femoral neck, a 37-year-old Caucasian woman was admitted via our emergency service with a painless, swollen finger. We diagnosed a fracture of the third proximal phalanx of her right hand based on the findings on physical and radiological examination and performed percutaneous pinning under local anesthesia. At follow-up six weeks later, she complained of swelling of her right foot. We observed three metatarsal fractures on X-ray examination and treated them conservatively.

**Figure 1:**
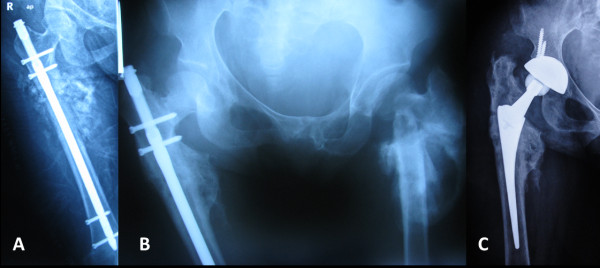
**Radiographic examination.** (**A**) Intramedullary nailing of a right subtrochanteric fracture. (**B**) Ipsilateral femoral neck fracture 18 months after nailing. (**C**) Cementless total hip arthroplasty.

Six months later, our patient presented with limping and edema of her left thigh. Radiographic evaluation resulted in the diagnosis of a Fielding type III subtrochanteric femur fracture with excessive callus formation; this was treated non-surgically, with bed rest [6]*.* Two weeks later, she again presented to our hospital, this time for right hip deformity and pathologic displacement after falling in the bathroom. Radiographs indicated a Fielding type I right subtrochanteric femur fracture without callus. We considered this a fresh fracture and performed intramedullary nailing under general anesthesia (Figure 1A). We observed a dropped foot at follow-up two months later due to excessive callus formation, which had entrapped the sciatic nerve. We resected the hypertrophic callus and freed the sciatic nerve.

Our patient did not attend any follow-up appointments over the next eighteen months, but then presented to our hospital with similar symptoms, including a swollen thigh and limping. The neurologic deficit in her right foot had resolved. We detected a right femoral neck fracture; intramedullary nailing had previously been performed for a subtrochanteric fracture of the same femur (Figure 1B). We performed cementless total hip arthroplasty after removal of the nail under general anesthesia (Figure 1C). As was true for previous operations, she required no analgesia postoperatively. There were no anesthetic or infectious complications. We allowed mobilization with crutches two days after surgery. At her most recent follow-up, six months after the surgery, her active hip flexion was 100°, abduction 40°, and external and internal rotation 20° without any instability, and she could walk with full weight bearing.

We made a thorough evaluation and established a definitive diagnosis of CIP on the basis of the characteristic findings of multiple perioral mucosal ulcers, shortening of her nails and acro-osteolysis, a prematurely aged facial appearance, an undersized skeletal structure and Charcot arthropathy of her right ankle. We also detected anosmia, insensitivity to temperature differences and mild intellectual disability (according to Cattell Culture Fair Intelligence Test and The Kent Inventory of Developmental Skills). She had no evidence of muscular weakness or sweating disorders. In an intradermal histamine test (histamine phosphate 0.05mg/mL or 1:1,000 dilution), the axon reflex response, pain, wheal and flare were all absent. Dermal biopsy specimens showed no pathologic changes. On neurologic examination, her cerebellar function, proprioception and muscle strength were normal, however both deep and superficial sensation was absent.

## Discussion

CIP, a rare condition, was first described by Dearborn in 1932 [1]*.* Although it is a neurological condition, many of the manifestations are orthopedic. In this condition, both sensory and autonomic neurons are affected. Because pain is a protective mechanism, the absence of a sense of pain results in an absence of protective reflexes. Consequently, severe orthopedic problems such as fractures, Charcot arthropathy, acro-osteolysis, avascular necrosis, osteomyelitis and joint dislocations are commonly seen [2-5]*.* The pathophysiology of CIP is not clearly understood.

Dyck categorized HSAN into five different types according to the mode of inheritance, clinical features, degree of autonomic nervous system abnormalities and specific molecular genetic abnormalities [2]. HSAN 1 is also known as sensory radicular neuropathy. In this mild form of HSAN, neuropathic symptoms typically manifest in the second to fourth decade. The lower limbs are commonly affected, foot complications being the characteristic feature of this condition. Plantar ulcers over the metatarsal heads, paronychia of the toes, soft tissue infection, osteomyelitis and bone resorption are common [2]*.* Sensory symptoms and deficits are predominant. HSAN 2, a recessively inherited congenital sensory neuropathy also known as Morvan’s disease, is the most severe form of HSAN. All four limbs and sometimes the trunk are affected. Characteristics of the disorder are disease onset in infancy or early childhood, absence of sweating in the acral regions, absence of tendon reflexes and pathologic fractures. Motor symptoms and deficits overshadow sensory ones. Intelligence is usually normal. HSAN 3 is known as familial dysautonomia or Riley-Day syndrome. The hallmarks of this disorder are premature death, a history of poor feeding, pulmonary infections due to vomiting, areflexia, corneal insensitivity, autonomic disturbances such as defective temperature control, defective lacrimation and hypertension. HSAN 4, a form of CIP with anhidrosis, is a severe form of HSAN. Its characteristics are mild intellectual disability, behavioral disturbances, defective temperature control mechanisms, normal muscular strength and tendon reflexes, and lack of sweating. The main pathology is a lack of skin innervation by A and C fibers. HSAN 5 is a mild form of CIP in which nociception is selectively affected [2]*.* According to Dyck’s categories, we classified our patient as HSAN I.

The anesthesia protocol for patients with HSAN is similar to that for the normal population. Many reports have stated that anesthesia can be achieved with standard doses of anesthetic agents. However, regardless of the type of the surgery, these patients do not require opioids postoperatively. Mild perioperative hypothermia is to be expected and can easily be managed by using warm blankets and adjusting the environmental temperature [5,7]*.* In one patient with HSAN 4, a 19-month-old child, severe bradycardia progressed to fatal cardiac arrest with the use of halothane [7]*.* This complication of halothane had previously been described [8]. In our case, we performed all operations under general anesthesia without incident. Our patient did not require opioids postoperatively.

Common orthopedic manifestations of CIP are delayed diagnosis of fractures, joint dislocations, limb deformities and leg length discrepancies due to malunions and growth disturbances, Charcot joints, acro-osteolysis, early callus formation, heterotrophic ossification, osteomyelitis, foot problems such as plantar ulcers, and lacerations and burns due to thermal insensitivity [2,3,5]*.* In our case, we observed multiple fractures at different times. On each presentation, our patient arrived in our emergency service unaccompanied, despite limping on several occasions, with bilateral subtrochanteric femoral fractures and a femoral neck fracture. On other visits, we detected metatarsal fractures with callus formation, multiple rib fractures and a phalangeal fracture. We also observed sciatic nerve entrapment after hypertrophic callus formation, wound ulcers, acro-osteolysis and a Charcot joint. We treated the different types of fractures with different treatment modalities. We managed the third proximal phalanx of her right hand by percutaneous pinning under local anesthesia. We managed her left subtrochanteric femoral fracture conservatively because of the delayed diagnosis; abundant callus had already formed. We diagnosed her right subtrochanteric femoral fracture early and treated it by intramedullary nailing. Eighteen months later, we diagnosed an ipsilateral femoral neck fracture.

The goal of treatment in patients with a femoral neck fracture is an early return to satisfactory functional status with minimal morbidity and mortality, while minimizing the need for re-operation. Her late presentation to our emergency service precluded the use of osteosynthesis treatment options. Because she had a long life expectancy, we performed cementless total hip replacement rather than hemiarthroplasty to avoid long-term wear of the acetabular cartilage, protrusion and the subsequent need for conversion of the hemiarthroplasty to a total hip replacement. A cementless total hip replacement should last a minimum of two decades. The most important point is the survival of the prosthesis.

Limitations of our study include the limited duration of follow-up and the lack of genetic studies due to unfavorable financial conditions.

## Conclusions

This case illustrates orthopedic manifestations and problems in a patient with CIP. There have been no previous reports of total hip replacement in patients with CIP.

All joints should be examined carefully in these patients, a neurologic consultation arranged, and the patients educated on the strategies for, and importance of, preventing further injuries. The value of regular follow-ups should be explained to the patients and their families. Attempts to improve medical knowledge in this field will provide a better understanding of the management of patients with CIP.

## Consent

Written informed consent was obtained from the patient for publication of this case report and accompanying images. A copy of the written consent is available for review by the Editor-in-Chief of this journal.

## Abbreviations

CIP, Congenital insensitivity to pain; HSAN, Hereditary sensory autonomic neuropathy.

## Competing interests

The authors declare that they have no competing interests.

## Authors’ contributions

ME, KB, YI, HHC and IT were involved in the conception, design and interpretation. ME and YI wrote the manuscript. KB, HHC and IT collected data, reviewed relevant published reports and provided the images. All authors read and approved the final manuscript.
